# Mechanosensitivity Is a Characteristic Feature of Cultured Suburothelial Interstitial Cells of the Human Bladder

**DOI:** 10.3390/ijms21155474

**Published:** 2020-07-31

**Authors:** Jochen Neuhaus, Andreas Gonsior, Sheng Cheng, Jens-Uwe Stolzenburg, Frank Peter Berger

**Affiliations:** 1 Department of Urology, Research Laboratory, University of Leipzig, Liebigstr. 19, 04103 Leipzig, Germany; chengsheng2005@zju.edu.cn; 2 Department of Urology, University Hospital Leipzig, Liebigstr. 20, 04103 Leipzig, Germany; andreas.gonsior@medizin.uni-leipzig.de (A.G.); jens-uwe.stolzenburg@uniklinik-leipzig.de (J.-U.S.); 3 Department of Urology, University Hospital Jena, Am Klinikum 1, 07747 Jena, Germany; frank.berger@med.uni-jena.de

**Keywords:** human urinary bladder, afferent signaling, mechanical stimulation, shear stress, fura-2 calcium imaging, intercellular coupling, functional syncytium, platelet derived growth factor receptor-alpha, calreticulin, vimentin, alpha-smooth muscle cell actin, connexin 43

## Abstract

Bladder dysfunction is characterized by urgency, frequency (pollakisuria, nocturia), and dysuria and may lead to urinary incontinence. Most of these symptoms can be attributed to disturbed bladder sensitivity. There is growing evidence that, besides the urothelium, suburothelial interstitial cells (suICs) are involved in bladder afferent signal processing. The massive expansion of the bladder during the filling phase implicates mechanical stress delivered to the whole bladder wall. Little is known about the reaction of suICs upon mechanical stress. Therefore, we investigated the effects of mechanical stimulation in cultured human suICs. We used fura-2 calcium imaging as a major physiological readout. We found spontaneous intracellular calcium activity in 75 % of the cultured suICs. Defined local pressure application via a glass micropipette led to local increased calcium activity in all stimulated suICs, spreading over the whole cell. A total of 51% of the neighboring cells in a radius of up to 100 µm from the stimulated cell showed an increased activity. Hypotonic ringer and shear stress also induced calcium transients. We found an 18-times increase in syncytial activity compared to unstimulated controls, resulting in an amplification of the primary calcium signal elicited in single cells by 50%. Our results speak in favor of a high sensitivity of suICs for mechanical stress and support the view of a functional syncytium between suICs, which can amplify and distribute local stimuli. Previous studies of connexin expression in the human bladder suggest that this mechanism could also be relevant in normal and pathological function of the bladder in vivo.

## 1. Introduction

The urinary bladder has a dual function, periodical stretching during the filling phase and exhibiting mass contraction for micturition. The realization of this requires a complex coordination with the sphincter systems of the bladder itself, called the vesical or internal sphincter (musculus sphincter vesicae) and the split external or urethral sphincter, comprising an internal smooth muscle part (musculus sphincter urethrae glaber) and an external striated part (musculus sphincter urethrae transversostriatus) [1]. While the neuronal control via spinal cord and higher central nervous system centers are well known [2], the local signal processing in the bladder wall is still enigmatic. Many details of the lower urinary tract function have been learned from diseased states in humans and in animal models, such as bladder outlet obstruction (BOO) and overactive bladder (OAB), and interstitial cystitis/bladder pain processing in the bladder wall is still enigmatic. Many details of the lower urinary tract function have been learned from diseased states in humans and in animal models, such as bladder outlet obstruction (BOO), overactive bladder (OAB), and interstitial cystitis/bladder pain syndrome (IC/BPS) [3,4]. While the urothelium acts as the major sensor of bladder filling, the suburothelial interstitial cells are emerging as important modulators of the afferent signal generation [5]. Twenty-eight transient receptor potential (TRP) channels have been described so far in mammals, of which TRPV1, 2, 4 (vanilloid), TRPM8 (melastatin), and TRPA1 (ankyrin) seem to play an important role in sensory signaling in the bladder [6]. Recently, our own group described TRPA1 expression in interstitial cells (ICs) of the human, guinea pig, and rat, supporting the hypothesis of ICs being mechanosensitive [7]. The role of the ICs in the bladder is still not fully understood and recent studies indicate that there are different subtypes of ICs. The major differences in their distribution define sublayers of the lamina propria, referred to as the upper lamina propria and deeper lamina propria [8]. Recent comparative studies on ICs in human and the major laboratory animals suggest that the understanding of the roles of ICs partly needs revision [7,9,10].

Coupling between ICs, the urothelium, and detrusor smooth muscle cells is another important issue which determines the normal function of the bladder and is significantly altered in disease, as demonstrated in BOO [11,12,13,14,15,16], OAB [17,18], and IC/BPS [19]. Furthermore, cell culture studies showed that cytokines can control connexin (Cx)43 and Cx45 expression in bladder smooth muscle cells and interstitial cells [20,21,22]. Besides the formation of functional syncytia via gap junction coupling, connexins and the closely related family of pannexins can form hemichannels in the plasma membranes, mediating, for example, cellular ATP release [23,24].

In the present study, we used fura-2 calcium imaging as a direct readout for (i) spontaneous Ca^2+^ activity, (ii) mechanical pressure stimulation, (iii) shear stress, and (iv) hypotonic stretch in cultured human suburothelial interstitial cells of myoid differentiation (suICs). We describe the Ca^2+^ peak morphology, time resolved intracellular traveling of the Ca^2+^ wave, and the signal propagation to neighboring cells.

## 2. Results

### 2.1. Spontaneous Ca^2+^ Activity in Cultured Human suICs

**Figure 1:**
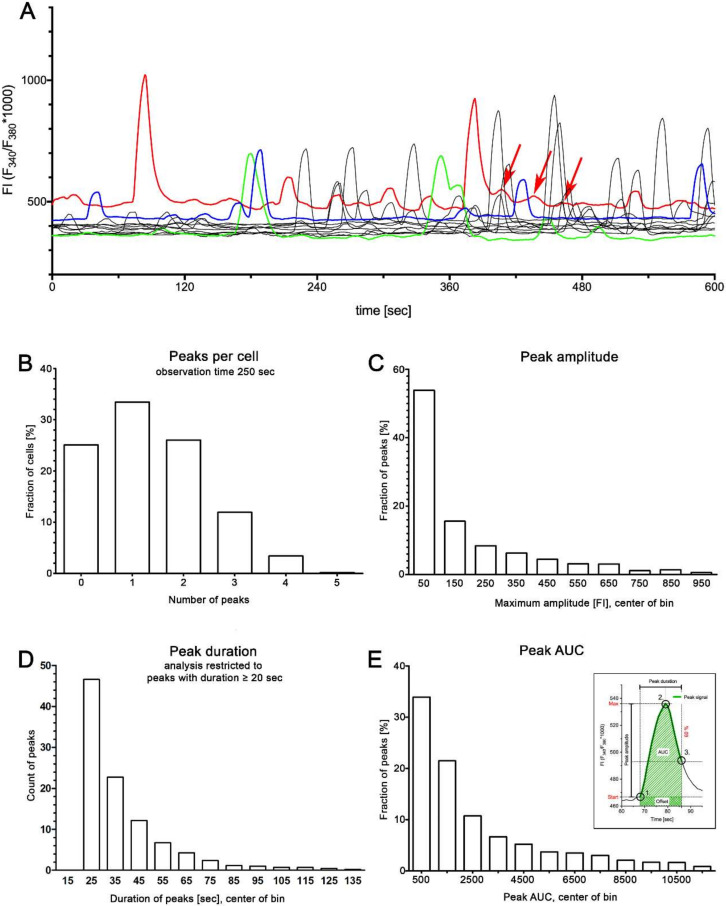
Spontaneous calcium activity in cultured suburothelial interstitial cells (suICs). (**A**) Representative traces of calcium transients measured with the ratiometric fura-2 fluorescent Ca^2+^ indicator; arrows indicate undulated decay of the amplitued in the red colored trace; (**B**–**E**) quantitative analysis of peak frequency (**B**), peak amplitude (**C**), peak duration (**D**) and integrated peak area (area under the curve = AUC (**E**); inset in (**E**) explains the peak measures (for detailed information, see Methods section).

**Figure 2:**
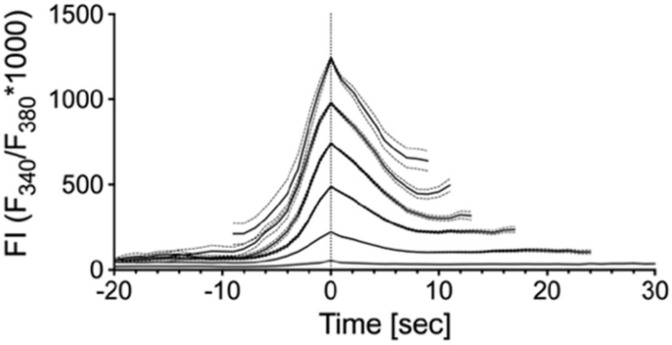
Peak morphology of spontaneous Ca^2+^ transients is homogeneous. Amplitude sorted transients were overlaid at their maximum. Note that the morphology does not alter with the amplitude. Mean (solid lines), standard error of the mean (SEM, dashed lines); only peaks with at least 20 time points were included.

After bulk loading of the cells with 2.5 mM of fura-2-acetoxymethylester (fura-2AM), spontaneous Ca^2+^ activity was recorded for 250 s (*n* = 11 experiments). In total, 1173 cells were analyzed. At least one transient elevation of the intracellular calcium concentration (calcium transients) was recorded in 879 (75%) of the cells, while 25% of the cells showed no spontaneous calcium activity (Figure 1A). Detailed analysis of the peak characteristics revealed a mean of 0.4 peaks /min (*n* = 879 cells, SD 0.21, 95% CI (0.42–0.45)). The mean peak amplitude was ΔFI = 200 (*n* = 1592 peaks, SD 262, 95% CI (188–214)) and the mean peak duration was 38.5 s (*n* = 1592 peaks, SD 23.8, 95% CI (37–40)). Notably, the area under the curves (AUCs) and the peak amplitudes varied considerably (Figure 1B–E). We found a strong positive correlation between the peak amplitude and the AUC (Spearman’s rho *r*_s_ = 0.959, *p* < 0.001) and a significant but very weak correlation between amplitude and peak duration (*r*_s_ = 0.045, *p* = 0.045). AUC weakly correlated with the peak duration (*r*_s_ = 0.267, *p* = 0.001). While the peaks varied in amplitude and frequency, their morphology was remarkably consistent; a fast rise of [Ca^2+^]_i_ followed by a slow signal decay (Figure 2) was observed. We observed no plateau phase, but the decay often undulated (Figure 1A, arrows).

To answer the question of whether the amplitude influences the overall shape of the peaks, we investigated the peak morphology by grouping the peaks according to their maximum amplitude into six groups (Table 1) and centering the transients to their maximum. We found that the peak shape was very similar at different amplitudes (Figure 2).

### 2.2. Single Cell Mechanical Stimulation by Glass Micropipette

**Figure 3:**
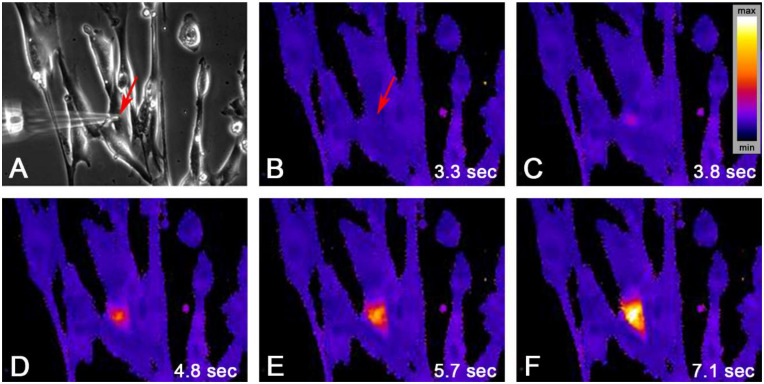
Positioning of the glass micropipette (**A**, phase contrast); lowering of the micropipette (**B**, arrow) provoked a local intracellular calcium rise (**C**) in the foremost quiet cell (arrow); the calcium signal spread across the cell within several seconds (**C**–**F**); arrow indicates micropipette tip position; inset in (**C**) = fluorescence intensity (FI) color-coding.

We analyzed 14 independent experiments (two cell cultures). Cells were bulk-loaded with fura-2AM, as described in Section 4.4. Ratio images (340 nm/380 nm) were acquired at a rate of 10 frames per second (fps) in seven experiments and at a rate of 4 fps in seven experiments. The fluorescence intensity (FI) was calculated after background correction as FI = F340 nm/F380 nm ×1000. The mean time of observation after initiation of the calcium signal was 128 s (range: 75 s–199 s). The FI was false color-coded (blue = min FI to white = max FI). An example of a typical experiment is depicted in Figure 3. A calcium transient was initiated in each of the 14 experiments. Compared to the spontaneous calcium transients, the transients had higher mean (±SD) amplitudes (2242 ± 967 FI vs. 599 ± 319 FI, *p* < 0.0001, unpaired *t*-test), larger AUCs (476737 ± 430178 vs. 41535 ± 37684, *p* < 0.0001, Mann–Whitney test) and lasted longer (40.99 ± 20.02 vs. 28.29 ± 14.13 s, *p* = 0.0363, Mann–Whitney test).

**Figure 4:**
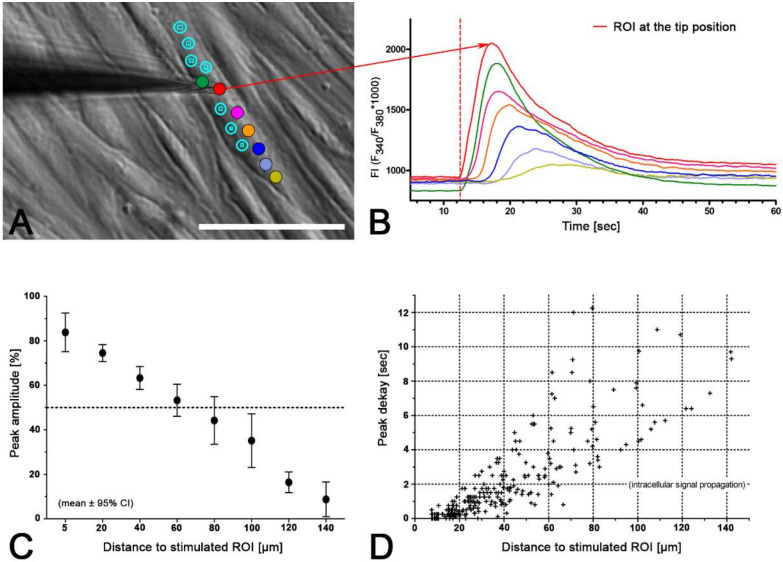
Intracellular propagation of the calcium signal. (**A**) Position of the glass micropipette (phase contrast): circles indicate the regions of interest (ROIs) of fluorescence measurements, color corresponds to the color-coding of the time resolved fluorescence intensity traces in (**B**); scale bar = 200 µm; (**B**) the fluorescence peak intensity decreased with increasing distance from the site of signal initiation; traces smoothed; (**C**) peak amplitude (%) versus distance to the site of stimulation (binned data, *x*-axis = center of bin; bin 5 = > 0 to < 10; bin 20 = ≥ 10 to < 30)—note the linear decay; (**D**) plotting of the peak delay versus the distance to the site of stimulation showed mainly linear correlation with increasing scattering at higher distances.

The calcium increase spread radially over the whole cell (Figure 4). The peak amplitude showed a linear decay by 50% at a distance of over 80 µm from the site of initiation (Figure 4C). The mean propagation velocity was 32.8 µm/s (*n* = 32, 95% CI = 12.9–52.6) for region of interest (ROI) distances up to 50 µm (Figure 4D, circles). At higher ROI distances, the velocity was slightly lower (mean = 23.2 µm/s, *n* = 120, 95% CI = 14.4–32.1); however, the difference was not significant (single-sided Mann–Whitney-Test, *p* = 0.25).

**Figure 5:**
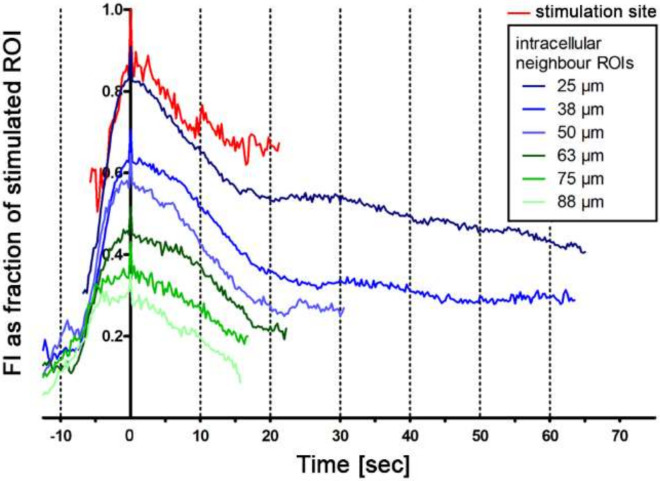
Peak morphology of calcium transients depending on the distance to the initial peak. Note that the peaks show similar rising and decay characteristics within 10 s before and after the peak maximum.

We evaluated the morphology of the peaks at different distances from the initial calcium signal by averaging peaks measured in seven experiments at 4 fps time resolution. The peak morphology was comparable in respect to signal rise and decay within the 10 s before and after the peak, respectively. However, with increasing distance from the initial peak, the curve flattened out. None of the cells reached the basic pre-stimulation calcium concentration within the observation time of 65 sec (Figure 5).

### 2.3. Intercellular Propagation of the Ca^2+^ Signal

**Figure 6:**
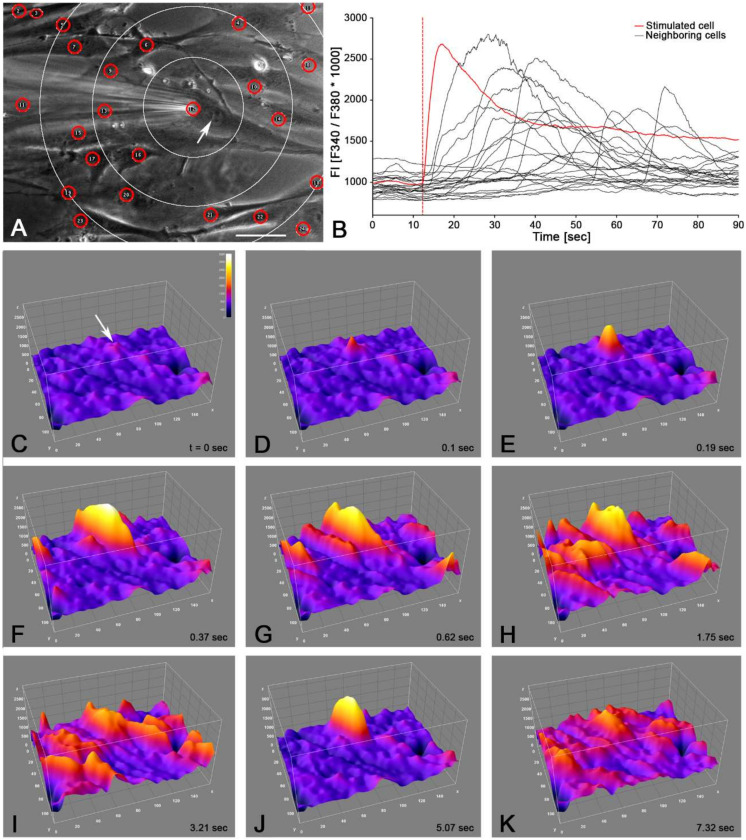
Analysis of the intercellular propagation of the calcium signal. (**A**) Position of the micropipette (stimulation site); ROIs (red circles); white circles indicate sections: 50 µm, 100 µm, 150 µm); nucleus of stimulated cell (white arrow); phase contrast image; scale bar = 50 µm. (**B**) Typical recording of calcium transients in the stimulated cell (red trace) and the neighboring cells (black traces); recording speed: 10 fps. (**C**–**K**) Surface plot of the propagation of the calcium wave over the functional syncytium: (**C**) pre stimulation; (**D**) 0.1 s after stimulation; white arrow indicates stimulation site. Note that first calcium peaks in neighboring cells already occurred within 1 s after stimulation (**E**–**G**); maximum excitation is seen about 3-5 s after stimulation (**I**,**J**); excitation decay is seen after 7 s (**K**); instant of time since stimulation is indicated bottom right; color-coding ranged from FI = 0–3200 (inset in **C**).

In our experiments, we observed a rise in calcium activity following single cell stimulation in neighboring suICs (Video S1). Therefore, we analyzed this phenomenon in 14 independent experiments (*n* = 2 cell cultures). The stimulated cell was placed in the center of the field of view after mechanical stimulation of the cell by lowering a glass micropipette onto the cell surface. The micropipette was retracted as soon as a calcium rise was observed in the cell. We recorded at least for 60 s at 4 fps (*n* = 7) or 10 fps (*n* = 7). Automatic analysis was done using a self-written Python 3 program [25]. The first ROI (stimulation site) was placed in the stimulated cell and further ROIs were placed in each neighboring cell center close to the nucleus (Figure 6). We calculated the radial distance of the neighboring cells (ROIs) to the stimulation site and grouped the ROIs (group 50: distance ≤ 50 µm etc.).

To reduce background noise, we filtered the peaks for a duration ≥ 10 s and an amplitude ΔFI ≥ 200. All the stimulated cells (14/14) showed a calcium transient. In each case, the first peak occurring in a cell was analyzed. Of the 604 neighboring cells examined, 40% showed a calcium peak within 60 s after stimulation. A representative experiment is depicted in Figure 6C–K.

**Figure 7:**
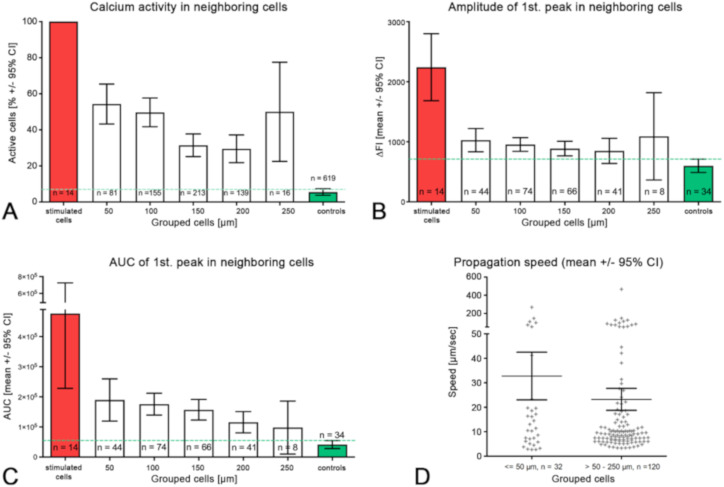
Analysis of the calcium peaks and transients in neighboring cells compared to the first peaks in the stimulated cells; (**A**–**C**) the neighboring cells were grouped into distance groups (upper bin limit indicated); (**D**) the propagation speed of the calcium signal was compared between near (≤50 µm) and far (>50–250 µm) neighboring cells. Note the split of the *y*-axis and the bimodal distribution of speeds; controls = cells with spontaneous activity; green dotted line indicates upper 95% CI of the controls.

With growing distance from the stimulation site, we found (i) a reduction in the fraction of active cells, (ii) a smaller peak amplitude, (iii) reduced duration of the calcium transient, (iv) diminished AUC and (v) an increase in the delay of the peak start (Figure 7, Table 2). Within a radius of 100 µm, 51.27% of the neighboring cells showed increased calcium activity, and within 250 µm, 39.24% of the neighboring cells showed increased calcium activity.

For peak analysis, we included only peaks which showed a higher amplitude compared to the spontaneous peaks measured before the stimulation experiment (control in Table 2). The threshold was defined as peak amplitude > 2 * standard deviation of the control. According to this criterion, only approximately 2.5% of the observed peaks should have been spontaneous.

The intercellular propagation velocity of the calcium wave was between 6.5 and 18.5 µm/s in the central 50% of the neighboring cells (median = 9.6, range from 2.7 to 465.9 µm/s). The mean velocity was 25.2 ± 50.4 µm/s, mean ± SD. There was no significant difference between the propagation velocity to close cells within the radius of 50 µm (32.77 ± 55.11 µm/s) or far cells within a distance of > 50 -250 µm (23.23 ± 49.11 µm/s) of the stimulated cell (Figure 7D).

### 2.4. Shear Stress Evoked Calcium Transients

In addition to the single cell mechanical stimulation, we applied physical deformation of the plasma membrane by changing the flow in the laminar perfusion chamber, generating shear stress. Important for the experimental setup was the habituation of the cells to a constant ringer flow of 1 mL/min for 10 min. In total, we analyzed nine experiments with *n* = 596 cells (*n* = 3 cell cultures).

**Figure 8:**
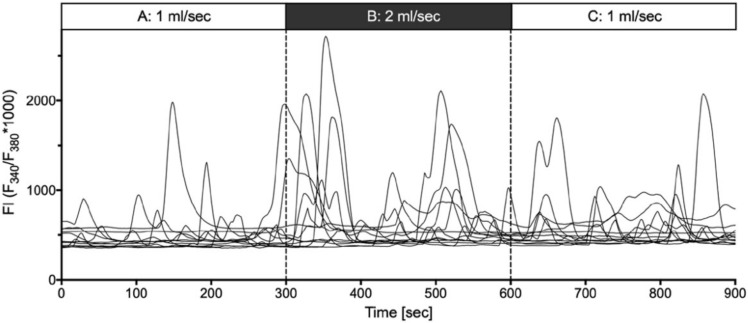
Effect of shear stress on the calcium activity of cultures suIC. In 5 min recordings, acceleration of the laminar flow from 1ml/s (**A**) to 2 mL/s (**B**) led to increased calcium activity. This activity decreased only slowly, as seen in the increased activity recorded 5 min after switching back to 1 mL/min flow (**C**).

The cells showed a significant increase in their calcium activity in section B, i.e., with increased laminar flow (Figure 8).

Interestingly, the reactions of the cells were not fully synchronized but there was a significant increase in the overall activity (peak frequency, amplitude, AUC and duration; Table 3).

### 2.5. Hypotonic Stimulation

**Figure 9:**
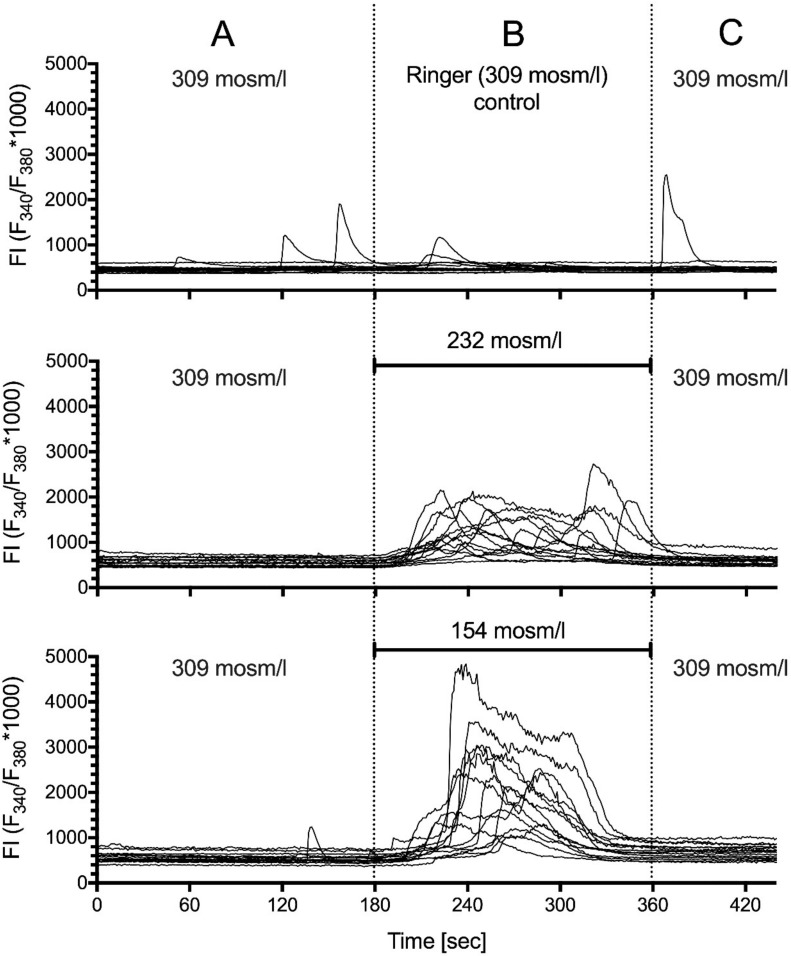
Effect of stimulation with hypotonic Ringer solutions. (**A**) conditioning of cells by 3 min preincubation in 309 mOsm/L Ringer; (**B**) 3 min period of challenging the cells with different Ringer solutions; Hypotonic Ringer led to a concentration dependent increase in calcium activity. This increase was almost synchronous and disappeared immediately after switching back to 309 mOsm/L Ringer solution (**C**). Representative traces of 14 cells per condition are depicted.

We used the hypotonic Ringer solution as a different method of mechanical stimulation as it leads to defined reversible swelling of the cells and stress on the cytoskeleton. The experimental setup corresponded to the stimulation by shear stress (see Section 2.4). In brief, the cells were adapted to standard Ringer (309 mOsm/L) at a flow of 1 mL/s for 15 min (Figure 9A). We recorded during the last 3 min of the preincubation. Then, the cells were challenged with either Hypo25 (232 mOsm/L) or Hypo50 (154 mOsm/L) for 3 min Figure 9B) followed by another 3 min recording in 309 mOsm/L Ringer (Figure 9C). We included control experiments with corresponding Ringer changes but using 309 mOsm/L in B and C. We analyzed a total of 111 cells in three independent experiments.

Figure 9 shows a representative experiment (14 cells of each condition included). We found a dramatic concentration dependent increase in the calcium activity elicited by hypotonic Ringer. The number of active cells increased from 5.9% in control Ringer to 100% under hypotonic stimulation; the peaks had a 22- (65-) fold higher amplitude and a 4.4- (5.5-) fold longer duration. This resulted in a 39-fold increase in 232 mOsm/L and a 93-fold increase in 154 mOsm/L in AUC (Table 4).

### 2.6. Immunocytochemical Characterization of Cultured suICs

**Figure 10:**
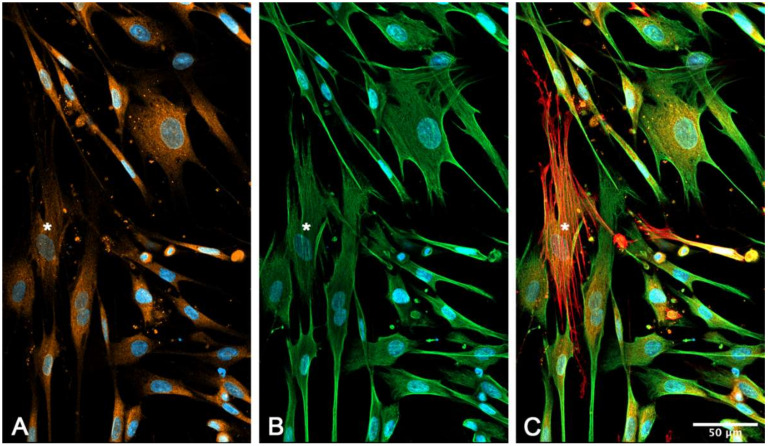
Immunocytochemical characterization of the suIC in cell culture. Triple staining of (**A**) calreticulin (CALR, orange), (**B**) vimentin (VIM, green) and (**C**) α-smooth muscle cell actin (aSMA, red); all cells show immunoreactivity (IR) for CALR and VIM, while only very few cells show strong aSMA staining of stress fibers (asterisks); nuclei are stained with 4′,6-diamidin-2-phenylindol (DAPI, blue).

**Figure 11:**
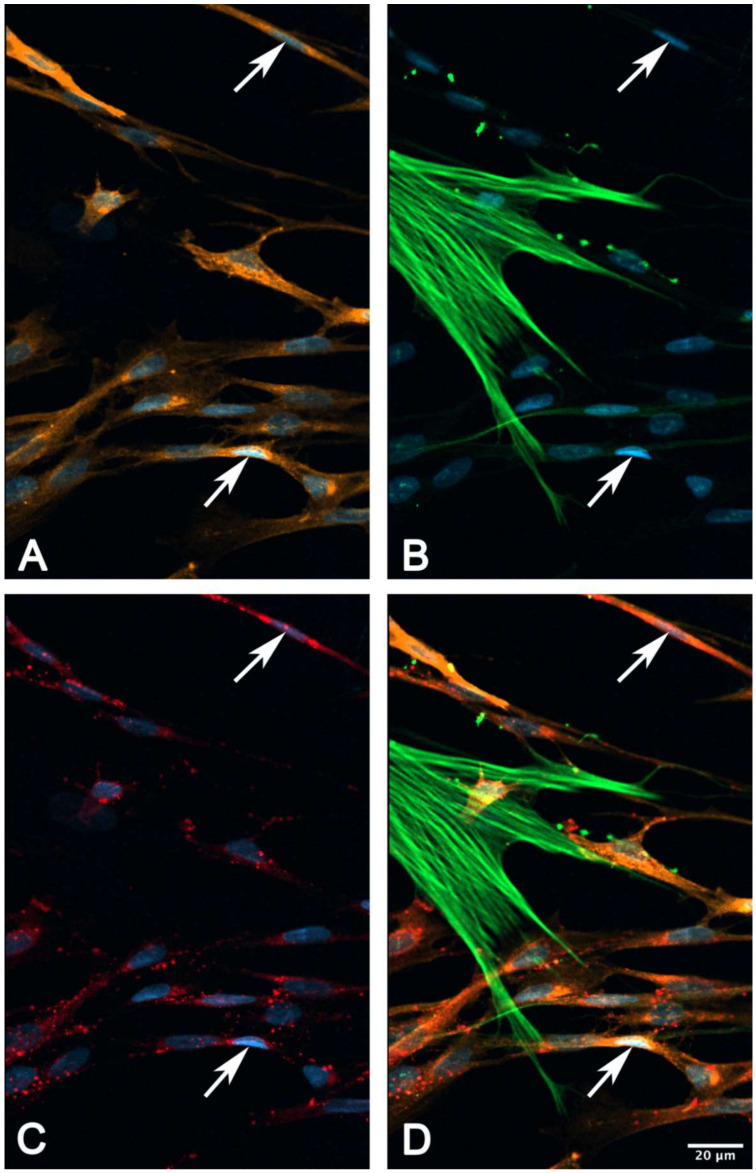
Triple staining of (**A**) platelet derived growth factor receptor alpha (PDGFRa, orange), (**B**) aSMA (green) and (**C**) connexin 43 (Cx43, red). Note the cell membrane associated punctuate or even patchy Cx43-IR present on cells with very small elongated nuclei and small cell bodies (arrows); no Cx43-IR can be attributed to the strongly stained myofibroblast characterized by the presence of stress fibers; nuclei are stained with DAPI (blue); (**D**) merged image channels.

We used cell cultures of passage 4-6 to examine the expression of characteristic marker proteins. We performed multiplex immunocytochemical detection of CALR/VIM/aSMA, PDGFRa/aSMA/CALR and PDGFRa/aSMA/Cx43 to define the suIC subtypes. All cells showed VIM-IR, classifying them as mesenchymal ICs (Figure 10B). Most cells were PDGFRa+/aSMA−/CALR+ with medium-sized ovoid nuclei and fusiform cell shapes (Figure 10 and Figure 11, Figure S2). A subpopulation was characterized by small elongated nuclei and at least one long process, which often extended to over 200 µm (Figure 10; Figure 11, arrow-heads). Only a small cell population (about 6%) showed classical myofibroblast differentiation, with strong aSMA staining of actin stress fibers, lacking PDGFRa expression (Figure 10, asterisks). In conclusion, most of the cultured suICs resembled the typical (type 1, PDGFRa+/CALR+/aSMA−) telocyte and a smaller fraction showed the characteristic staining pattern of hybrid (type 2, PDGFRa+/CALR+/aSMA+) telocyte, as defined by Vannucchi and coworkers [26].

In triple immunostaining experiments, we found that Cx43-IR was associated with PDGFRa+/aSMA+ or aSMA− cells. Cx43-IR was significantly lower in myofibroblasts (*p* = 0.01842, two-sided *t*-test, data not shown) in typical myofibroblasts showing extensive stress fibers (Figure 11).

## 3. Discussion

We here demonstrated for the first time that mechanical stimulation of cultured human suburothelial interstitial cells (suICs) evoked intracellular calcium transients. This observation is relevant for the assumed function of the suICs within the afferent signaling pathway in the bladder [27,28,29]. The current view is that the primary sensor of bladder filling—i.e., stretching of the bladder wall—is the urothelium, and it releases adenosine triphosphate (ATP), acetylcholine (ACh), nitric oxide (NO), prostaglandins (PGs), cytokines and many others involved in signal transduction to the ICs and nerve fibers in the lamina propria [30,31].

**Figure 12:**
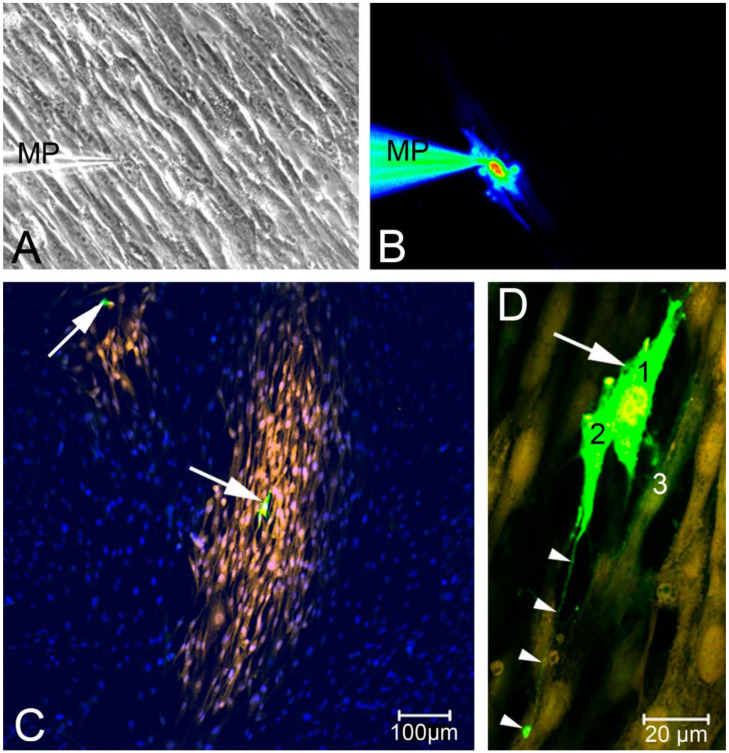
Lucifer Yellow (LY) injection revealed functional syncytia in cultured suICs. (**A**) Phase contrast, MP (micro pipette); (**B**) injected cell filled with LY after 90 s; (**C**) cell culture with two injected cells (arrows) showing distribution of the LY, defining variably sized functional syncytia; (**D**) the LY injected cell (arrow) and a direct neighboring cell show bright LY-fluorescence signal; arrow-heads indicate a fine process contacting another cell, spanning a distance of approximately 100 µm; nuclei (DAPI, blue); modified reproduction with permission from Neuhaus et al. 2007 [38].

We observed calcium transients in three different conditions:

- (i) Spontaneous calcium transients occurred regularly in the cultured human suICs and were confirmed in previous in vitro and in vivo studies in rats, guinea pigs and humans [29,32,33,34,35].
- (ii) Mechanical stimulation of human suICs either by direct indentation of the plasma membrane, swelling of the cells by hypotonic Ringer solution or membrane deformation by sheer stress induced calcium transients. Interestingly, calcium transients were also elicited when releasing the sheer stress by reduction of the flow rate to normal flow. This indicates that the cells are also capable of sensing the changing of the pressure in addition to the force of the pressure.
- (iii) Calcium signals propagated not only intracellularly but were transmitted to neighboring cells. This indicates that the suICs formed a functional syncytium, as previously shown [21,22]. Interestingly, we observed two populations of neighboring cells, with approximately 87% of the cells showing a delay of calcium activation after single cell stimulation, resulting in an intercellular signal propagation velocity of about 20 µm/s, while in 20 of 152 cells (13%), the delay was much shorter, with a calculated propagation speed of ≥ 50 µm/s. These cells were similarly found in close proximity to the stimulated cell (<50 µm radius), as well as within a distance of 50 to 250 µm (Figure 7D). We hypothesized that the latter cells were directly connected to the stimulated cell via long thin cell processes, thus resembling telocytes [36], which also have been proposed to be present in the upper lamina propria (ULP) of the human bladder [26,37]. This idea was directly supported by dye coupling experiments, regularly demonstrating cells with long cytoplasmic processes in cultured suICs (Figure 12), and our immunocytochemical experiments indicating the presence of typical type 1 telocytes (PDGFRa+/CALR+/aSMA−) and of PDGFRa+/CALR+/aSMA+ hybrid (type 2) telocytes (Figure 10, Figure 11, Figure S2). We found type 1 telocytes, which were characterized by very small nuclei and small cell bodies showing long thin bipolar cell protrusions spanning up to 200 µm. These cells were observed at a rate of 12.5% in early cell cultures and decreased to around 7% with increasing confluency. Since we used the cell cultures at ≥ 80% confluency (e.g., Figure 4), it is most probable that we did not directly stimulate these cells. However, the small fraction of typical telocytes would well correspond to the observed 13% of cells with short Ca2+ signal delay in up to 250 µm distance (Figure 7D), thus promoting a fast calcium wave distribution over the cellular syncytium. Interestingly, we found no Cx43-IR in typical myofibroblasts accounting for less than 1% of the cells. These very large cells, therefore, would not be considered to contribute to the propagation of the calcium signal.

The intercellular propagation velocities of 32.8 µm/s and data from the literature promote the idea that IP3 rather than Ca^2+^ proper is responsible for the triggering of calcium transients in neighboring cells. IP3 has an up to 22-times higher diffusion rate and a 10-fold higher lifetime and is thus able to trigger Ca^2+^ release from neighboring cells in a range of 24 µm from the point of initiation [39,40]. In addition, there is evidence that Ca^2+^ rapidly closes GJ, possibly via binding to calmodulin (CaM), which in turn closes the GJ channel by binding to Cx43 [41]; review [42].

Interestingly, the propagation velocities of the calcium waves in the cell cultures were well below the velocities reported by Kanai et al. in bladder wholemounts of normal rats (4700 ± 700 µm/s) [29]. However, in contrast to the single cell stimulation in our cell culture experiments, the Ca^2+^ waves in the bladder wholemounts were initiated by stretching of the whole bladder preparation. As we have demonstrated in our hypotonic experiments, stretch can significantly enhance the spontaneous activity of suIC. Therefore, the cells in the wholemount experiments were pre-stimulated when calcium activity rose at a single site of the active bladder dome, facilitating the rise of [Ca^2+^]_i_ in neighboring cells and the Ca^2+^ wave propagation. In addition, synergisms of several signaling mechanisms in vivo—e.g., gap junction coupling, mechanical coupling and fast short-range sensitization of Ca^2+^ entry channels by the release of soluble activators—could further enhance the Ca^2+^ wave propagation, as indicated by the higher conduction velocities measured after carbachol stimulation [29].

**Figure 13:**
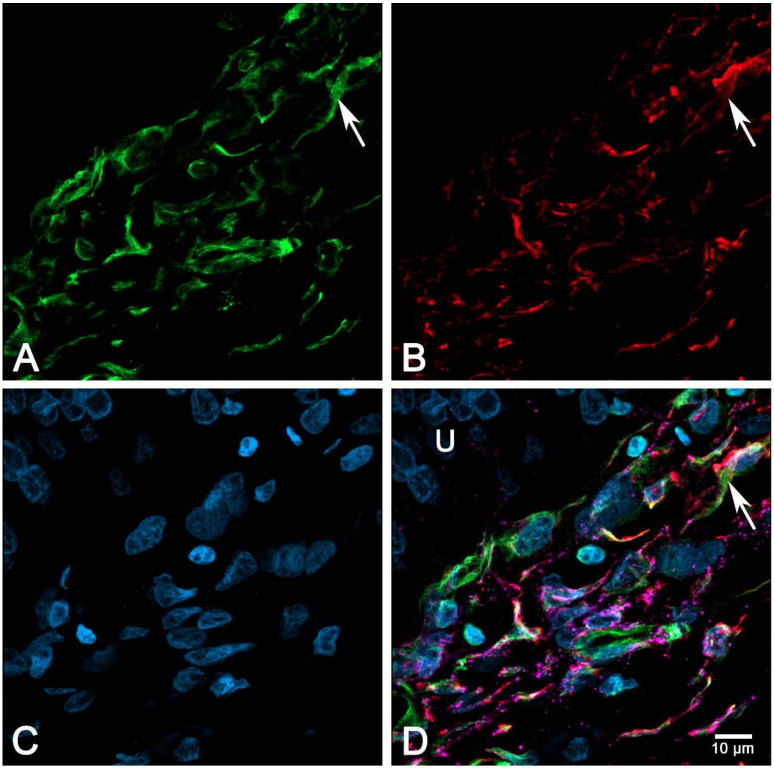
The 3D projection of a confocal image stack (voxel size (*yxz*): 0.06 µm × 0.06 µm × 0.39 µm); (**A**) VIM (green, ex 488 nm); (**B**) aSMA (red, ex 640 nm); (**C**) nuclei stained with DAPI (blue, ex 405nm); (**D**) merged image including Cx43 staining (magenta, excitation at 561 nm). Note that only a few cells are negative for aSMA (arrow); U = urothelium.

In a previous study, we found that the same cell cultures used in the present study expressed Cx43 and Cx45, which were differentially regulated by cytokines and growth factors, modulating intercellular coupling [21]. To study the morphology and the Cx43 expression of suICs in situ and in vitro, we used immunohistochemical multiplexing. The interstitial cells directly adjacent to the urothelium presented small cell bodies with two or more processes. Most of them showed a strong expression of Cx43, the molecular correlates of intercellular coupling. The majority of the cells in the ULP stained positive for the cytoskeletal markers vimentin (VIM) and alpha-smooth muscle actin (aSMA), as depicted in the confocal 3D projection (Figure 13, Supplementary Video S2).

Our study provides further evidence for a mechanism of non-neuronal signaling in the human bladder by a network of mesenchymal interstitial cells [5]. The relevance of these cells for the physiologic and pathologic function of the bladder has been implied by evidence at several levels. Their strategical positioning at the border between the urothelial layer and the deep lamina propria is accompanied by characteristic receptor expression [7,9] and by the expression of Cx43, leading to the formation of a functional syncytium in vivo [43].

In the present study, we focused on calcium transients in suICs activated by various mechanical stimuli. Though we did not directly show the involvement of TRPA1 channels in the mechanical activation of calcium transients, it is well documented in the literature that extracellular calcium is essential for eliciting intracellular calcium transients and intracellular calcium oscillations [44,45,46]. Arora and colleagues described how the graded stretch activated slow calcium oscillations in human gingival fibroblasts. These oscillations depended on extracellular [Ca^2+^]_e_, actin cytoskeleton and pertussis toxin-sensitive subunits of G-proteins but not on intracellular calcium pools, indicating the involvement of stretch-activated ion channels [46].

Calcium waves arise from massive calcium release from intracellular calcium stores upon activation of inositol triphosphate receptors (IP_3_Rs) and ryanodine receptors (RyRs) interacting with various members of the TRP family [45,47,48]. In the presence of extracellular Ca^2+^ and the selective TRPA1 agonists allyl isothiocyanate (AITC) and 15-deoxy-delta-12, 14-prostaglandin J2 (PGJ2) induced intracellular calcium transients in human cardiac fibroblasts, indicating the triggering of intracellular Ca^2+^ release via extracellular Ca2+ influx [44].

Additional support for the hypothesis of suICs being active elements in afferent signaling processing in the bladder comes from experiments demonstrating increased ATP release from cultured pig bladder ICs by hypotonic stimulation [49]. Our own group has previously shown that human suICs (denoted myofibroblasts in older literature) are highly sensitive to ATP, inducing calcium transients at very low concentrations [33].

In our single cell stimulation experiments, we did not observe any correlation between the direction of the Ringer flow with the spreading of the calcium wave in neighboring cells. Therefore, we conclude that a possible release of ATP from the locally stimulated suIC does not add to the calcium signal measured in our experiments and that the distribution of the signal is truly mediated via the cytoplasmic coupling of the cells.

Cultured suICs express aSMA, allowing cell contraction, especially in the fraction of myofibroblast-like cells forming stress fibers (Figure S1). However, our immunocytochemical experiments indicated that these myofibroblast-like cells are not included in the functional syncytium of the suIC under cell culture conditions. Besides strong aSMA expression, the typical fibroblast-like appearance of this cell—i.e., its large flat cell body with large nuclei and multiple cell processes—has provoked the name “myofibroblast”. Under normal conditions, such cells should not account for the majority of the aSMA+ ICs in the bladder. Therefore, these cells might indeed represent pathological altered suICs and it will be interesting to further investigate their contribution to pathological states of the bladder.

Since most non-myofibroblast suICs (type 2 telocytes) strongly express aSMA in situ and a rise in intracellular calcium can trigger cell contraction, it is conjecturable that local contractions evoked by ATP signaling from the urothelium could also lead to local signaling during the bladder filling phase. Therefore, the role of suICs in afferent signaling could be the enhancement and distribution of local signals over larger areas of the bladder wall. This view is supported by the excellent experiments of Kanai and colleagues in wholemount rat bladders, directly demonstrating the coordinated spreading of local (spontaneous) calcium signals from the dome to the bladder neck. Furthermore, they demonstrated the propagation of the signals from the mucosal initiation site to the serosa of the bladder [43].

If the suICs take part in afferent signal processing in the bladder, one should assume that local stimulation of a single cell, as was conducted in Section 2.2, should influence the calcium activity in the functional syncytium. We therefore compared the activity after stimulation in the neighboring cells with the approximated background activity measured in control experiments. We found that, after mechanical stimulation of a single cell, the syncytial activity (activity in the neighboring cells) increased by 18 times based on the AUCs in the first peaks. The primary calcium signal evoked by a single short-term membrane indentation of a single cell was radially distributed in the cell layer for at least 250 µm, activating an area of approximately 0.2 mm^2^. The strength of activation decreased with distance from 500% to 200% in a radius of 50 µm and 250 µm, respectively. In total, the primary calcium signal was amplified by 50%. These findings strongly support the notion of an amplifier function of suICs.

The major limitation of this study is the use of a 2D adherent cell culture system. In addition, while we provide some evidence of several subtypes of cells in our cell culture, we did not further characterize these subtypes to allow a more detailed view of the roles of these cells in the bladder. Since the urinary bladder wall is composed of multiple cell types embedded in a complex structured extracellular matrix, the results cannot be transferred directly to the in vivo situation. Physiological stretching of the bladder wall during bladder filling results in multiple cellular interactions, including the release of transmitter substances and mechanical stress. Therefore, the 2D model system can only highlight a small part of the complex sequence of events.

In the future, it will be interesting to investigate the role of suIC functional syncytia in 3D model systems of overactive bladder and especially of interstitial cystitis/bladder pain syndrome (IC/BPS).

## 4. Materials and Methods

### 4.1. Ethical Statement

This study was approved by the Ethics Committee of the University of Leipzig (ethical approval code: 036-2007, Date of issue: 26.05.2015) and was conducted according to the principles expressed in the World Medical Association Declaration of Helsinki [50]. Written informed consent was obtained from all patients.

### 4.2. Cell Cultures

Human bladder tissue was collected from patients undergoing cystectomy for bladder carcinoma treatment or due to gynecologic tumors. We were able to culture suICs from five female and three male patients, aged (mean ± SD) 60.2 ± 11.7 yrs and 64.3 ± 6.6 yrs, respectively. Cell cultures of suburothelial interstitial cells (suICs) were established from macroscopic tumor free bladder tissue by sharp microsurgical dissection of the urothelium from the smooth muscle layer of the detrusor, ensuring no contamination with detrusor smooth muscle cells. The tissue was then cut into small fragments of approximately 0.5 mm edge length and cultured in smooth muscle cell growth medium 2 (PromoCell GmbH, Heidelberg, Germany). This medium did not support the growth of urothelial cells and the resulting cell cultures were free of contaminating urothelial cells, as proven by polymerase chain reaction (PCR), Western blot and immunocytochemistry. We described the procedure in detail in a previous study [21]. In brief, the fragments were plated into tissue culture flasks (TPP AG, Trasadingen, Switzerland) and incubated at 37 °C in 5% CO_2_ humidified atmosphere until reaching 80% confluency. Cell passages 2 to 5 were used in the experiments. For the calcium imaging experiments, the cells were plated onto collagen A (Biochrome AG, Berlin, Germany) coated 13 mm glass cover slips and grown to 80% confluency.

### 4.3. Chemicals and Solutions

We used a modified Krebs–Ringer solution for the calcium imaging experiment (in mmol/L): CaCl_2_ 1.9; NaCl 120.9; NaHCO_3_ 14.4; KCl 5.9; MgCl_2_ 1.2; NaH_2_PO_4_ 1.55; Hepes 4.2; Glucose 11.49; pH = 7.2; 309 mOsm/L (all chemicals from Sigma-Aldrich, Steinheim, Germany). All solutions were freshly prepared with cell culture grade distilled water on the day of usage. For hypotonic solutions, we diluted the Krebs–Ringer solution at the ratio of 3:1 and 1:1 to yield 232 mOsm/L and 154 mOsm/L solution, respectively.

### 4.4. Calcium Imaging

Cells cultured onto glass cover slips were washed 3 times with Krebs–Ringer solution at 37 °C and thereafter incubated for 35 min at 37 °C with 2.5 mMol/L fura-2 AM (fura-2 acetoxymethylester) dissolved in DMSO (dimethylsulfoxid) and 2% pluronic (Invitrogen by Thermo Fisher Scientific, Schwerte, Germany). The cells were washed again in Krebs–Ringer solution and placed into a 37 °C heated lamina flow Diamond Bath Oocyte Recording Chamber (RC-26Z, Warner Instruments, Hamden, CT, USA). To exclude calcium activity caused by the washing and transfer procedure, the cells were adapted to the experimental conditions (37 °C, oxygenation of the Krebs–Ringer solution with 95% O_2_ and 5% CO_2_, constant flow of 1 mL/min) for ten minutes before the start of the experiments. We used an IX-71 inverted microscope equipped with a LCPLN20xIR objective (Olympus Microscopy, Hamburg, Germany) and a TILL IMAGO-QE digital camera (TillPhotonics, Gräfelfing, Germany). Experimental control and image acquisition were done using TILLvisION Software V. 4.5 (TillPhotonics). Image series were recorded at 1, 4 or 10 frames per second (fps). Fura-2 fluorescence was excited by 30 ms pulse application of 340nm and 380nm monochromatic light (Polychrome V monochromator, TillPhotonics) and the fluorescence signal was detected at a wavelength of 520 nm. The specific fluorescence intensity (FI) was calculated after subtraction of the background fluorescence: FI = F340 nm/F380 nm × 1000. The FI was color-coded and exported as TIFF video. We used a self-written Python [25] program for analysis of the calcium changes over time (calcium transients). The core of the Python program was the automatic calcium peak detection, which was fitted to cope with the heteromorphic calcium traces. The analysis can be restricted to pre-defined parts of the curves, allowing precise analysis of the peak morphology. For the analysis of intercellular calcium waves, the software allows the definition of a reference (stimulated) cell and calculates the distances of all regions of interest (ROI) and the time course of the calcium signal in relation to this cell. Details are described in the Supplementary Materials.

### 4.5. Data Analysis and Statistics

We used Microsoft Excel (Microsoft Corporation, Redmond, WA, USA) and Graph Pad Prism 5/8 (GraphPad Software, San Diego, CA, USA) for data processing and statistical analysis (Wilcoxon matched-pairs signed rank test; two-tailed Mann–Whitney test).

### 4.6. Stimulation Experiments

As a starting point, spontaneous calcium activity was recorded after 10 min of adaptation of the cells before starting the real experiment. We investigated the cells’ reactions to three different mechanical stimuli.

#### 4.6.1. Single Cell Mechanical Stimulation

**Figure 14:**
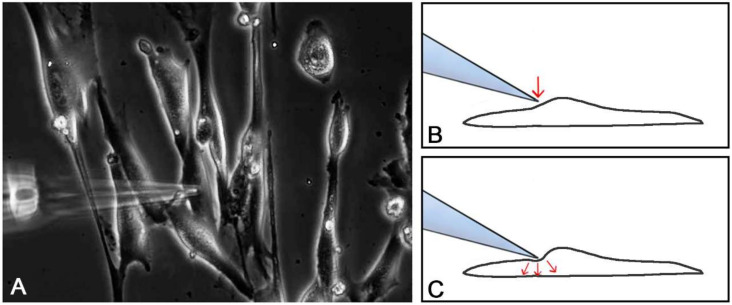
Mechanical stimulation using a glass micropipette. The micropipette is placed close to the plasma membrane (**A**); by stepwise (40 nm steps) lowering of the micropipette, the plasma membrane is dented until a calcium signal is initiated (**B**,**C**); red arrows indicate movement of the micropipette (**B**) and the deflection of the cell membrane (**C**).

Single cell mechanical stimulation requires ultraprecise micromanipulators. We used a motorized MP-285 Nano-Micromanipulator (Sutter Instruments, Novato, CA, USA), allowing step sizes down to 40 nm. We mechanically stimulated single cells by defined deflection of the plasma membrane using a glass micropipette with a fine tip, which was closed and rounded to avoid (i) capillary flow-in of ringer and (ii) mechanical injury of the membrane. The membrane deflection was applied by stepwise lowering the micropipette in steps of 40 nm (Figure 14).

#### 4.6.2. Mechanical Stimulation by Shear Stress

Cells were adapted to the experimental conditions by 15 min superfusion with 1 mL/min. We then recorded the calcium activity for 5 min in each section: (A) 1 mL/min, (B) 2 mL/min, (C) 1 mL/min.

#### 4.6.3. Hypotonic Stimulation

After adaptation for 15 min to the experimental conditions (309 mOsm/L, 1 mL/min flow), cells were challenged either with 232 mOsm/L or 154 mOsm/L for 3 min. Recordings of the calcium activity were done for three minutes in each section (A,B,C). For control, the flow was switched to 309 mOsm/L in section B.

### 4.7. Dye-Coupling Experiments

Single cells were dialyzed for 10 min with 3% lucifer yellow (LY) dissolved in intracellular pipette solution (130 mM/L KCl, 5 mM/L K-pyruvate, 5 mM/L K-oxalacetate, 5 mM/L K-succinate, 10 mM/l HEPES, 0.02 mM EGTA, pH 7.4) [51] by using a 50 MΩ micropipettes for sharp penetration of the cell membrane. LY was injected into the cell by short reproducible pressure pulses by using a PV800 Pneumatic PicoPump (World Precision Instruments, Berlin, Germany), as described previously [52]. LY-fluorescence was visualized using a fluorescein isothiocyanate (FITC) filter set.

### 4.8. Confocal Imaging

Confocal images of bladder tissue were acquired at a LSM800 (Carl Zeiss Microscopy, Jena, Germany) equipped with a Plan-Apochromat 63x/1.40 Oil DIC M27; in cell cultures, a 40x/0.95 objective was used for acquisition of 5 × 5 large tiles, which were stitched to yield an image of 556 µm × 556 µm. In brief: human bladder tissue was fixed in 4% buffered formalin for 24 h and embedded in paraffin. Ten µm thick paraffin sections were immunofluorescence stained for VIM, aSMA, Cx43 (Table 5). Cell cultures were cultured onto collagen A covered glass coverslips. Cells were fixed for 2 min in –20 °C methanol before processing for primary antibody incubation overnight at 4 °C. Nuclei were stained with DAPI (4′,6-diamidin-2-phenylindol, 1:5000, Carl Roth, Karlsruhe, Germany). Image processing and analysis was done in ImageJ 1.51s [53] for mac and Adobe Photoshop CC V. 20.0.10 (Adobe, San José, CA, USA).

## Figures and Tables

**Table 1:** Maximum amplitudes of the peak groups.

Group	Mean	SD	*N*
1	56.5466	27.46916	1913
2	221.3514	70.75953	582
3	488.9613	72.17626	245
4	743.6639	69.71185	134
5	978.3084	66.73132	70
6	1239.981	66.15836	29

SD = standard deviation; *N* = number of peaks analyzed.

**Table 2:** Number of cells observed and number of peaks analyzed.

Cell Group	0	50	100	150	200	250	Control
Distance (µm)	Stimulated	<= 50	>50 ≤ 100	>100 ≤150	>150 ≤ 200	>200≤ 250	NA
Number of cells	14	81	155	213	139	16	619
Active cells (*n*)	14	44	77	67	41	8	34
Mean (%)	100	54.3	49.7	31.5	29.5	50	5.5
95% CI (%)		43.2–65.4	40.3–50.2	25.2–37.7	21.8–37.2	12.9–51.6	3.7–7.3
Peak ampl. mean	2242	1025	954	887	848	1090	599
95% CI	1683–2800	831–1219	840–1068	766–1008	639–1058	363–1817	489–711
Peak AUC (au)	476737	189641	175652	157005	115,647	97981	41535
95% CI	228,359–725,115	119,516–259,766	139,339–211,966	122,889–191,121	80,430–150,865	9981–185,982	28,387–54,684
Peak duration (s)	40.9	36.8	36.8	33.4	28.1	39.3	28.3
95% CI	29.4–52.6	32.8–40.9	34.1–39.6	30.5–36.3	24.6–31.7	19.4–39.2	23.4–33.2
Peak delay (s)	NA	6.534	11.65	18.34	18.1	15.68	NA
95% CI		4.1–8.9	9.4–13.9	15.4–21.3	12.9–23.3	10.1–21.3	

NA = data not available; control = spontaneous peaks before stimulation.

**Table 3:** Analysis of the shear stress experiments.

Peak	Section A	Section B	Section C	*p*–Value
Frequency (min^−1^)	0.62 (522)	0.70 (554)	0.75 (559)	A/B: <0.0001 §
	[0.59–0.64]	[0.68–0.73]	[0.72–0.78]	B/C: 0.0039 §
Amplitude (FI)	269.4 (1345)	344.7 (1628)	207.6 (1757)	A/B: <0.0001 $
	[245.6–293.19]	[319.4–370.0]	[192.1–223.2]	B/C: <0.0001 $
Duration (s)	34.26 (1345)	39.43 (1628)	35.74 (1757)	A/B: <0.0001 $
	[32.99–35.54]	[38.03–40.83]	[34.59–36.90]	B/C: <0.0001 $
AUC	5789 (1345)	8860 (1628)	4602 (1757)	A/B: <0.0001 §
	[5149–6430]	[8060–9660]	[4172–5032]	B/C: <0.0001 §

Mean (*n*), [95% CI]; § Wilcoxon matched pairs signed rank test; $ two-tailed Mann–Whitney test.

**Table 4:** Analysis of the hypotonic stimulation experiments.

Osmotic Conc.	309	232	154	*p*–Value
(mOsm/L)	Control	Hypo25	Hypo50	
frequency (min^–1^)	0.02 (44)	0.45 (36)	0.35(58)	Hypo25/Hypo50
	[0.0–0.06]	[0.39–0.49]	[0.34–0.36]	<0.0001 $
amplitude (FI)	32.63 (1)	733.2 (47)	2147 (59)	Hypo25/Hypo50
	[NA]	[579.8–886.7]	[1891–2403]	<0.0001 $
duration (s)	21 (1)	93 (47)	114.6 (59)	Hypo25/Hypo50
	[NA]	[80.19–105.8]	[109.4–119.8]	0.0021 $
AUC	419.6 (1)	37,707 (47)	119,702 (59)	Hypo25/Hypo50
	[NA]	[27,896–47,517]	[102,882–136,421]	<0.0001 $

In control, only one cell showed spontaneous activity; mean (*n*), [95% CI]; $ two-tailed Mann–Whitney test.

**Table 5:** Primary and secondary antibodies used in confocal imaging experiments.

Target	Host Isotype	Class	Conjugate	Dilution	Product Number	Source
vimentin (VIM)	mouse gG1	Monoclonal	na	1:200	V6389	a
alpha-smooth muscle actin (aSMA)	mouse IgG2a	Monoclonal	na	1:1000	A2547	a
platelet-derived growth factor receptor-alpha (PDGFRa)	goat	Polyclonal	na	1:100	AF-307-NA	b
calreticulin (CALR)	rabbit	Polyclonal	na	1:600	ab2907	c
connexin 43 (Cx43)	rabbit	Polyclonal	na	1:500	C6219	a
mouse IgG1	goatIgG	Polyclonal	Alexa Fluor® 488	1:500	A21121	d
mouse IgG2a	goat IgG	Polyclonal	Alexa Fluor® 633	1:500	A21136	d
rabbit IgG	goat IgG (H+L)	polyclonal	Alexa Fluor® 555	1:500	A21121	d
mouse IgG	donkey IgG (H+L)	monoclonal	Alexa Fluor® 488	1:500	A21202	d
goat IgG	donkey IgG	polyclonal	Alexa Fluor® 555	1:500	A41232	d
rabbit IgG	donkey IgG	polyclonal	Dylight® 649	1:500	711-495-152	e

(a) Sigma-Aldrich (Taufkirchen, Germany); (b) R&D Systems, Mineapolis, MN, USA; (c) Abcam, Cambridge, MA, USA; (d) Invitrogen by Thermo Fisher Scientific, Schwerte, Germany; (e) Dianova, Hamburg, Germany; na = not applicable.

## Supplementary Materials

Supplementary materials can be found at https://www.mdpi.com/1422-0067/21/15/5474/s1.

## Abbreviations

AUC	area under the curve
aSMA	alpha-smooth muscle actin
BOO	bladder outlet obstruction
CALR	calreticulin
Cx43	connexin 43
DAPI	4′,6-diamidin-2-phenylindol
FITC	fluorescein isothiocyanate
fps	frames per second
fura-2AM	fura-2-acetoxymethylester
IC/BPS	interstitial cystitis/bladder pain syndrome
OAB	overactive bladder
PDGFRa	platelet derived growth factor receptor-alpha
SD	standard deviation
SEM	standard error of the mean
suIC	suburothelial interstitial cells
TRP	transient receptor potential
VIM	vimentin
