# Supplementary material for: Mechanosensitivity Is a Characteristic Feature of Cultured Suburothelial Interstitial Cells of the Human Bladder
Source: Int J Mol Sci. 2020 Jul 31;21(15):5474. doi: 10.3390/ijms21155474 (PMC7432121; doi:10.3390/ijms21155474)
Supplement: Supplementary file 1 [file ijms-21-05474-s001.zip › Figures S1 and S2.docx]

# Appendix A


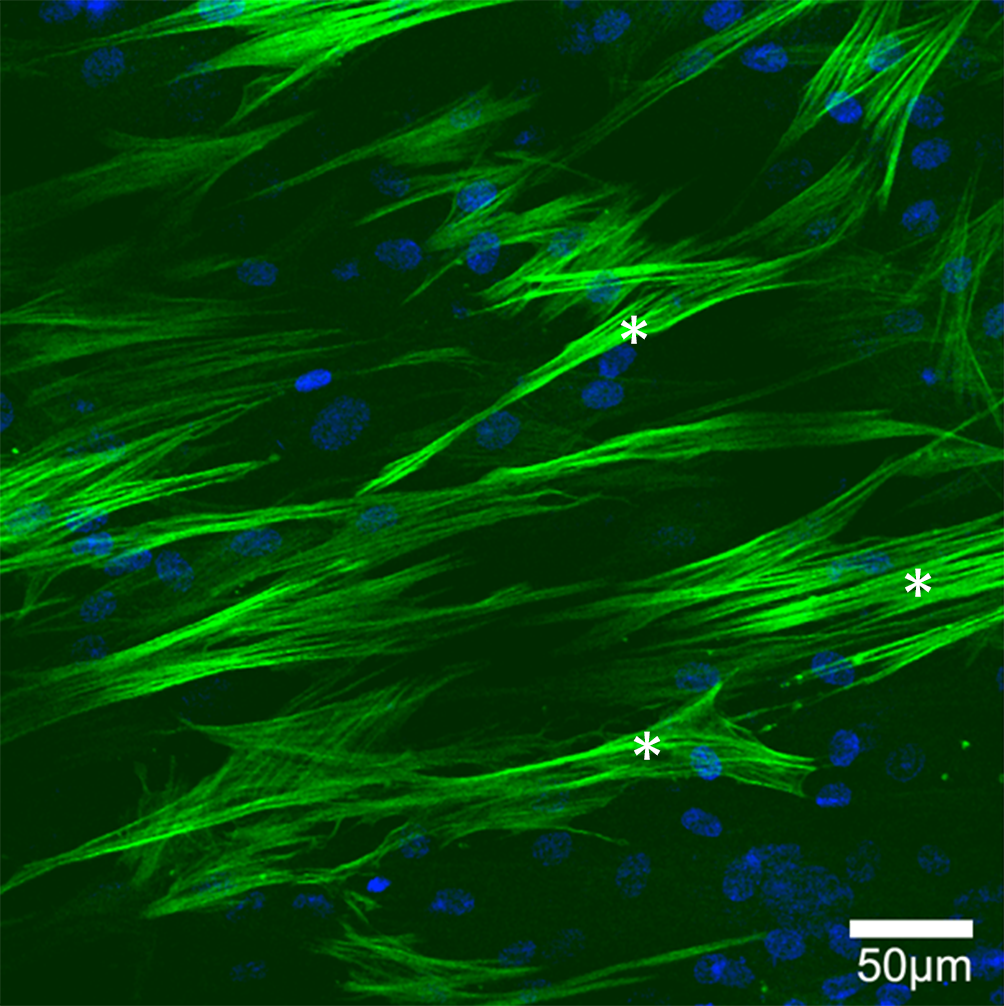


**Figure A1.** Confocal image of aSMA (green) expression in suICs. A fraction of suICs show the formation of stress fibers (asterisks); nuclei stained with DAPI (blue).


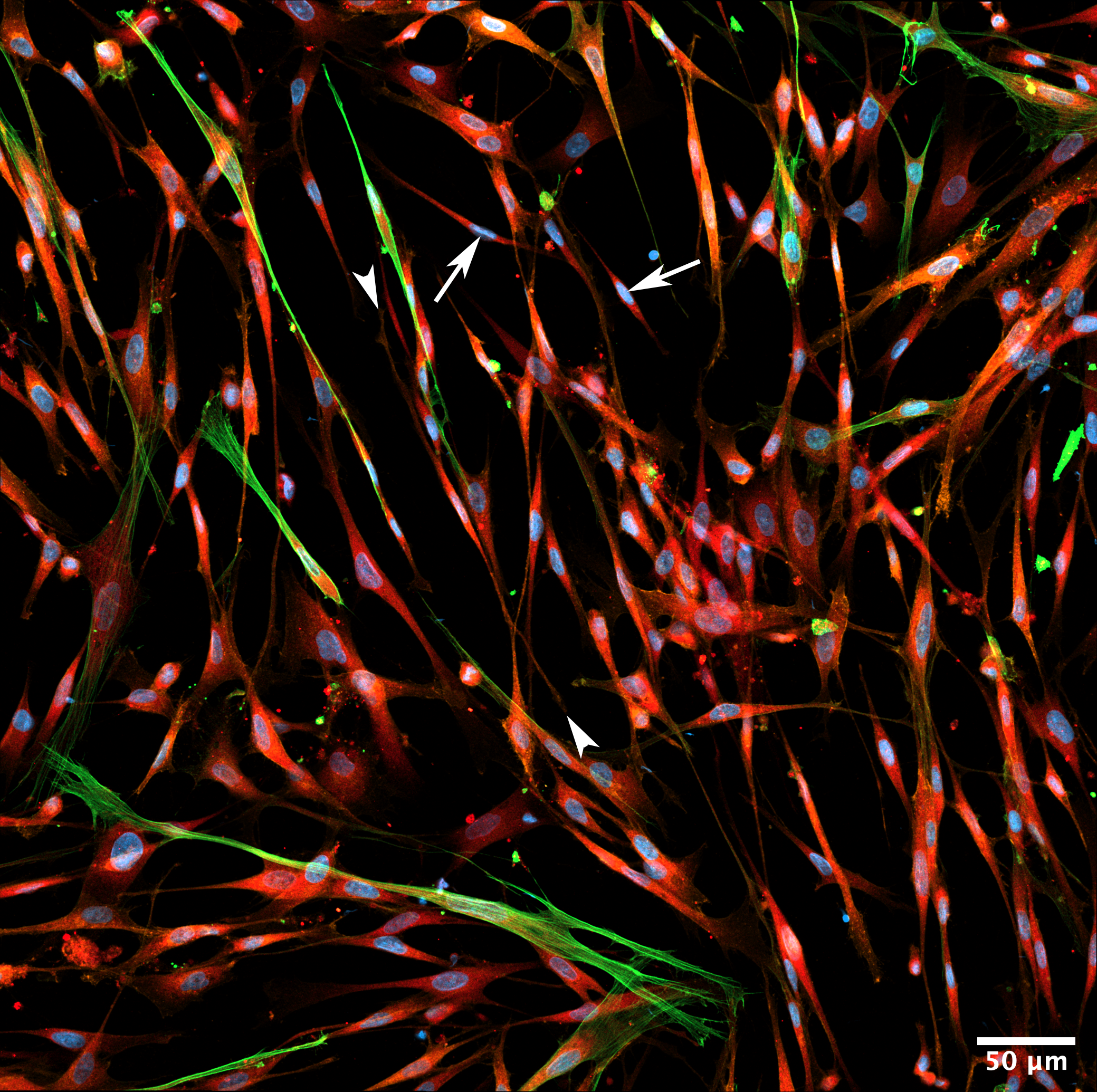


**Figure A2.** Confocal image of a 60 % confluent suIC cell culture triple stained for PDGFRa (orange), aSMA (green) and CALR (red); nuclei stained with DAPI (blue); cells with very small elongated nuclei are PDGFRa+ / CALR+ but aSMA- (arrows); those cells show very thin bipolar cell processes reaching a length of about 200 µm (arrow heads).
