# Supplementary material for: Mechanosensitivity Is a Characteristic Feature of Cultured Suburothelial Interstitial Cells of the Human Bladder
Source: Int J Mol Sci. 2020 Jul 31;21(15):5474. doi: 10.3390/ijms21155474 (PMC7432121; doi:10.3390/ijms21155474)
Supplement: Supplementary file 1 [file ijms-21-05474-s001.zip › Supporting Methods.docx]

# **Supplemental Methods**

Peak analysis:

1. Automatic peak detection

The analysis of the peaks involved the automatic detection of the peaks by definition of a threshold depending on the noise level in the individual cell.

1.1. Filtering

We applied a mean filter to reduce high-frequency noise. We used the same smoothing factor for all data (SF=5; fluorescence ratio (FI) = F340 / F380):


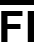
 (n) = (FI(n-2) + FI(n-1) + FI(n) + FI (n+1) + FI(n+2)) / 5


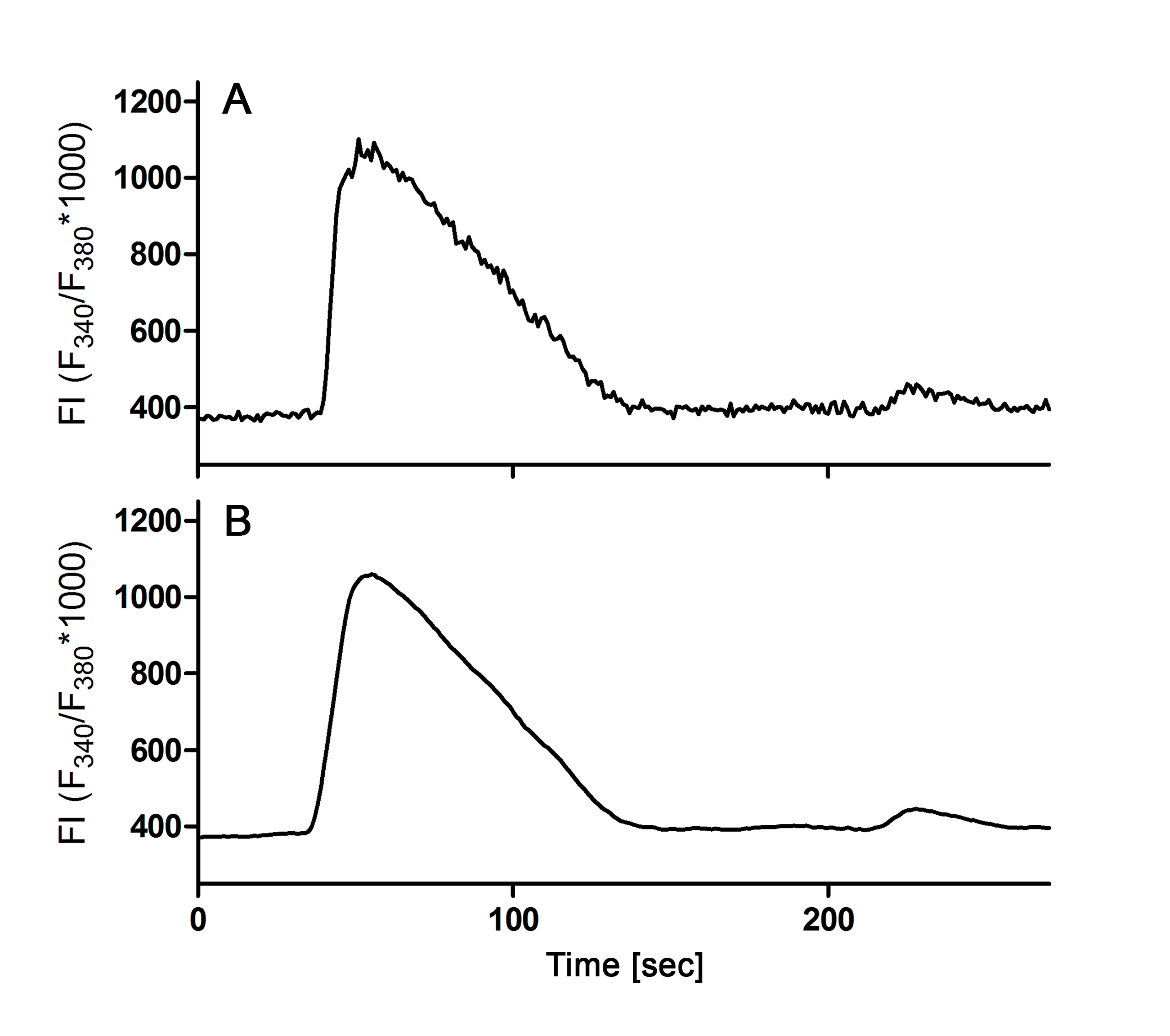
 **Fig. 1.** (A) original recording trace; (B) trace after smoothing.

1.2. Detection of a reference background signal

The mean FI of 20 consecutive data points with minimal amplitude were used as a reference for individual background noise.


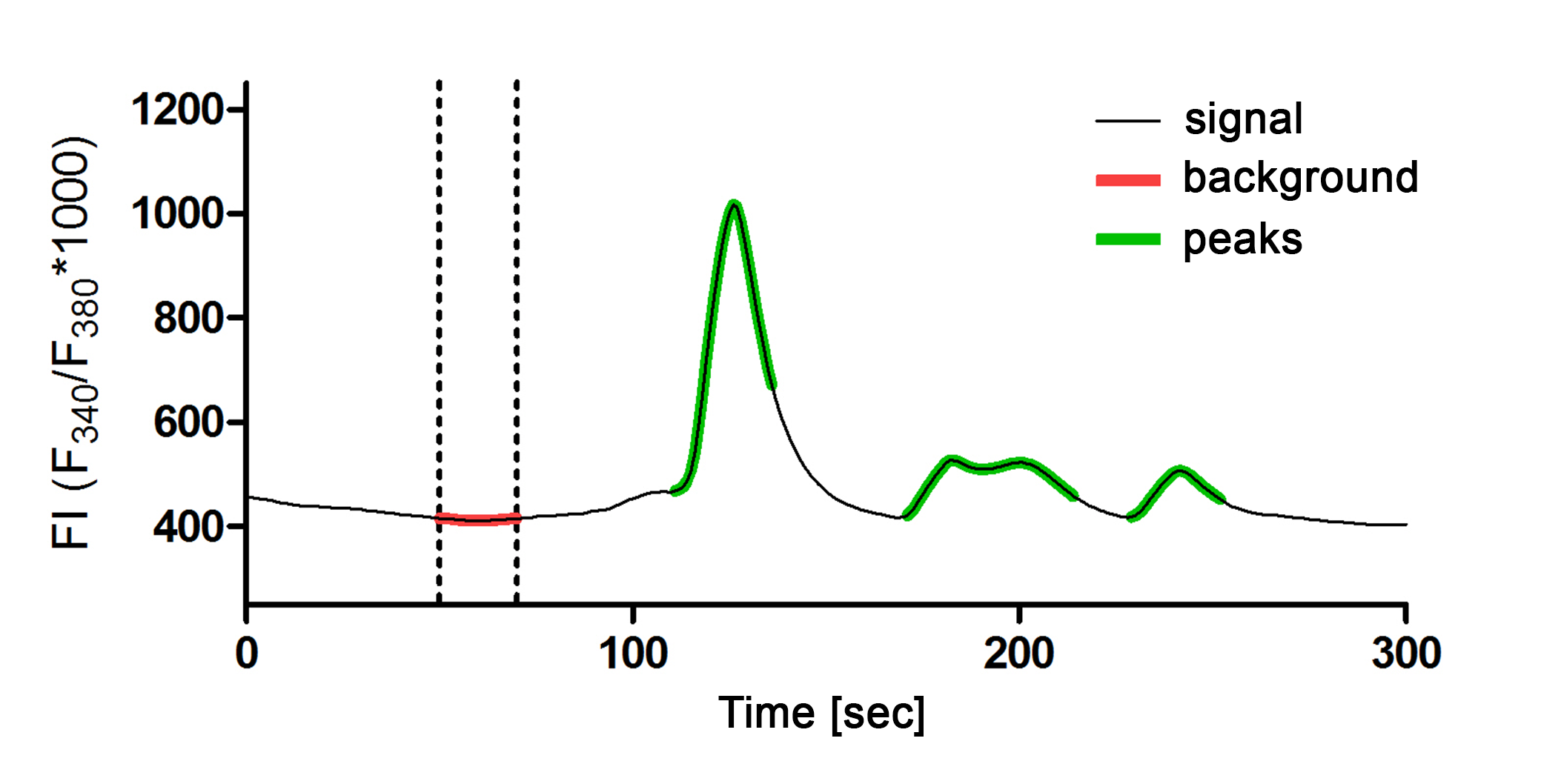


**Fig. 2.** The background FI is defined as the mean FI of 20 consecutive datapoints with minimal amplitude (red); detected peaks (green).

1.3. Detection of the start of a peak

The threshold (∆Fl/dt) for the definition of a signal was defined as 2.5-times of the standard deviation of the background signal.

1.4. Definition of the end of the peak

From the calculated peak amplitude (PA = FImax – FImin) based on the start of the peak threshold (1.3.), we defined the end of the peak (PE) at a decay to 40% of the PA.

1.5. Minimum amplitude and minimum duration of a peak

Only peaks with a minimum amplitude of ∆FImin = 20 and a minimum duration of 1 second were included in the analysis.

**
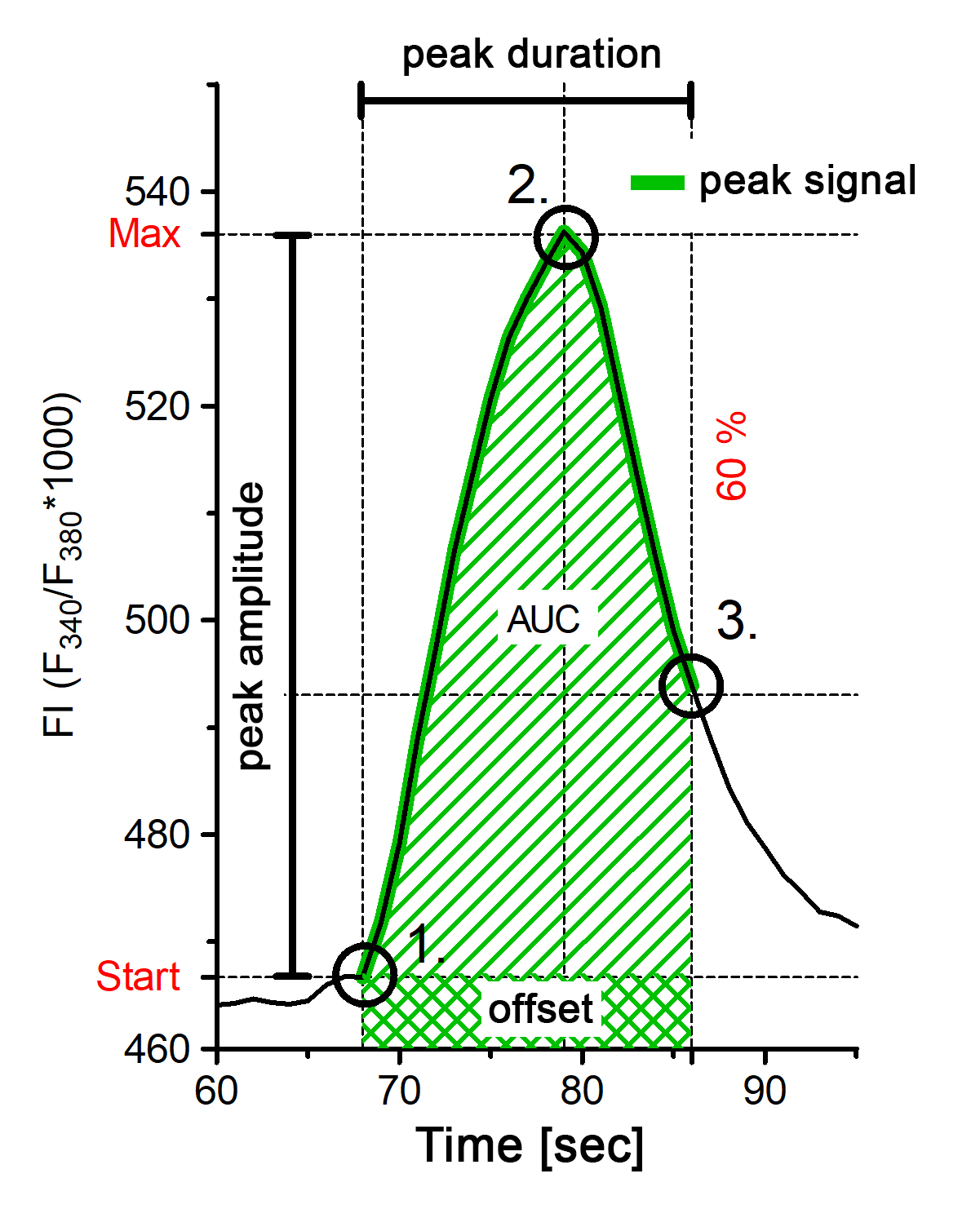
**1.6. Parameters analyzed

**Fig. 3.** Peak characterization. (1.) peak start (FI_start_); (2.) peak maximum (FI_max_); (3.) peak end (FL_end_); AUC: area under the curve (hatched), offset: area below FI_start_ (cross-hatched).
